# Adaptation reconceptualized: "retrofitting" ongoing organizational activities with essential elements of evidence-based interventions

**DOI:** 10.1186/1748-5908-10-S1-A35

**Published:** 2015-08-20

**Authors:** Mary Janevic, Tyra Bryant-Stephens, Marielena Lara, Yvonne Ohadike, Victoria Persky, Gilberto Ramos-Valencia, Shelley Stoll, Kimberly Uyeda, Floyd Malveaux

**Affiliations:** 1 Department of Health Behavior and Health Education, University of Michigan, Ann Arbor, MI 48109, USA; 2 Children's Hospital of Philadelphia, Philadelphia PA, 19104, USA; 3 Rand Corporation, Santa Monica, CA 90401-3208, USA; 4 Merck Childhood Asthma Network, Washington, D.C., 20004, USA; 5 Division of Epidemiology and Biostatistics, University of Illinois at Chicago, Chicago IL, 60607, USA; 6 Department of Biostatistics and Epidemiology, University of Puerto Rico, San Juan, Puerto Rico 0093, USA; 7 Student Medical Services, Los Angeles Unified School District, Los Angeles, CA 90017, USA

## Background

Evidence-based interventions (EBIs) are sometimes implemented in contexts where ongoing program activities overlap with EBI components. As part of a comprehensive cross-site evaluation, we examined the grant-funded implementation of the EBI "Yes We Can" (YWC), a medical-social model for pediatric asthma care coordination, in four distinct settings. Assessment indicated that some sites already had in place one or more activities that were similar to YWC components. We examined why and to what extent organizations "retrofitted" existing activities to align with YWC core elements, and how retrofitting influenced implementation and maintenance.

## Methods

YWC essential elements were identified through a literature search and interview with the developer. We obtained information about ongoing organizational activities and subsequent adaptations from in-depth interviews with site leaders and field staff, in which factors affecting implementation [1] were also probed. Detailed interview notes were coded and analyzed, using NVivo.

## Results

The extent of retrofitting ranged from one extreme, where the EBI was adopted where no similar activities existed, to the other, where a well-established, successful program was improved by adopting select components of YWC. Key informants noted the value of keeping "what already works" in their unique context while enhancing ongoing services by adding EBI components. Factors affecting implementation highlighted the conditions that make retrofitting a rational and feasible approach; e.g., a flexible, complex intervention that allows for significant adaptations and the opportunity to capitalize on existing political support for program activities. In this small sample, sites that retrofitted ongoing activities were better able to maintain EBI components after funding ended.

## Conclusion

Retrofitting reflects the reality of how some EBIs are adapted, and may promote successful maintenance. Future research can explore systematically how retrofitting affects other key outcomes such as adoption, reach, and effectiveness.

Supported by the Merck Childhood Asthma Network.
